# Same habitat types but different use: evidence of context-dependent habitat selection in roe deer across populations

**DOI:** 10.1038/s41598-018-23111-0

**Published:** 2018-03-23

**Authors:** Gaudry William, Gaillard Jean-Michel, Saïd Sonia, Bonenfant Christophe, Mysterud Atle, Morellet Nicolas, Pellerin Maryline, Calenge Clément

**Affiliations:** 1 Office National de la Chasse et de la Faune Sauvage, Unité Ongulés Sauvages. Direction de la Recherche et de l’Expertise, 85 bis Avenue de Wagram, 75017 Paris, France; 2 Centre National de la Recherche Scientifique (CNRS), Unité Mixte de Recherche (UMR) 5558, Laboratoire de Biométrie et Biologie Évolutive, Université de Lyon, Université Lyon 1, 43 boulevard du 11 novembre 1918, 69622 Villeurbanne, France; 3 Centre for Ecological and Evolutionary Synthesis (CEES), Department of Biosciences, University of Oslo, P.O. Box 1066 Blindern, NO-0316 Oslo, Norway; 4 INRA, UR35 Comportement et Ecologie de la Faune Sauvage, Institut National de la Recherche Agronomique, CS 52627 31326 Castanet-Tolosan, France

## Abstract

With the surge of GPS-technology, many studies uncovered space use of mobile animals and shed light on the underlying behavioral mechanisms of habitat selection. Habitat selection and variation in either occurrence or strength of functional responses (i.e. how selection changes with availability) have given new insight into such mechanisms within populations in different ecosystems. However, linking variation in habitat selection to site-specific conditions in different populations facing contrasting environmental conditions but the same habitat type has not yet been investigated. We aimed to fill this knowledge gap by comparing within-home range habitat selection across 61 female roe deer (Capreolus capreolus) during the most critical life history stage in three study areas showing the same habitat types but with different environmental conditions. Female roe deer markedly differed in habitat selection within their home range, both within and among populations. Females facing poor environmental conditions clearly displayed a functional response, whereas females facing rich environmental conditions did not show any functional response. These results demonstrate how the use of a given habitat relative to its availability strongly varies in response to environmental conditions. Our findings highlight that the same habitat composition can lead to very different habitat selection processes across contrasted environments.

## Introduction

Habitat selection is a hierarchical process involving behavioral decisions made by an individual about what habitat should be used relative to those available at different spatial and temporal scales^1,2^. Traditionally, resource selection studies have compared the use of a resource with its availability at a specific spatial (i.e., food item, resource patch, home range, geographic distribution)^3^ and temporal scale^4^ (i.e., second, day, week, month, year, lifetime), inferring a selection for or against a particular resource type when the use is not proportional to the availability^5,6^. Although recent technological progress allows to monitor animal movement continuously^7^ and thereby analysing habitat selection by considering the full continuous range of spatial scales, instead of targeting a limited number of pre-defined spatial scales, the four main hierarchical orders of selection defined by Johnson^3^ are still intensively used for its heuristic value. Two selection scales have received a particular attention: the landscape scale (2^nd^ order) that consists of comparing home range composition to landscape composition and involves dispersal or seasonal migration processes, and the home range scale (3^rd^ order) that consists of comparing the time spent in each habitat type within the home range to its composition and involves foraging and resting behaviors at the daily scale^8^.

At the home range scale, animals should match the relative use of different habitat types with their current vital needs according to the resources available in those habitat types (e.g. “foraging habitat” providing food vs. “cover habitat” providing protection against predators)^9^. Thus, animals are likely to face trade-offs like, for example, risk-related trade-offs, and habitat selection is the result of these trade-offs within their home range^10,11^. Because it affects the costs to benefits ratio of selecting a given habitat, the shape and the intensity of these trade-offs can vary in response to changes in the relative availability of habitats providing different essential resources^12,13^. Such a change in the strength of habitat selection according to habitat availability corresponds to a functional response in habitat selection^12^. Considering functional response in habitat selection is of prime importance as it can ultimately affect population dynamics. Indeed, recent studies have demonstrated that the behavioral response of individuals to variation in resource availability yields different fitness payoffs^14,15^.

Empirical evidence indicates that the shape of functional responses varies among populations of large herbivores depending on the local trade-off situation^11,13^. When animals face a food vs. cover trade-off, a decrease in the availability of foraging habitats should increase the strength of selection for these habitats at the expense of cover habitats, which leads to a negative functional response^10,12,16^. Red deer (*Cervus elaphus*) increased selection for pasture (forage habitat) at the expense of forest (cover habitat), when the availability of pasture decreases within individual home ranges^10^. On the other hand, when animals face a forage quantity-quality trade-off, the value of a given habitat should increase with its availability, leading to a positive functional response^13^. Reindeer (*Rangifer tarandus*) increasingly selected habitats with high quality forage over habitats with high biomass when the relative availability of habitats with high quality forage increases^13^.

The strength of functional response has also been reported to vary across seasons^10^ or among populations facing contrasted levels of resource availability^11^. Godvik *et al*.^10^ found that the functional response differs across seasons between two red deer populations facing different levels of pasture and forest availability. During summer, the functional response is strongest in the study area characterized by high forest availability but poor pasture availability, whereas the reversed pattern is observed in spring, as the functional response is stronger in the study area with high pasture availability. Thus, interacting effects between season and landscape habitat composition might affect the trade-offs experienced by animals, generating differences in the strength of functional responses. Marked differences in the occurrence and in the strength of functional responses might even occur between populations very close spatially^11^.

Such variation in the occurrence and strength of functional responses among populations facing different environmental conditions suggests that the relationship between the use of a habitat and its availability can be influenced by local environmental conditions^10,11^. Indeed, the foraging decision made by an individual depends on the availability of alternative resource^17^. Thus, considering the environmental context (i.e. main limiting factors such as climate, resource quality, presence of predators) in which a resource is available is of prime importance to assess reliably space use patterns of individuals. However, to our knowledge, no study has yet assessed comparatively across populations how use of the same habitats even after controlling for availability may differ depending on the context, which limits our understanding of the mechanisms involved in habitat selection. We aim to fill this knowledge gap by comparing within-home range habitat selection among adult female roe deer (*Capreolus capreolus*) during the critical period of their life cycle (i.e., in spring and summer when female energy expenditures peak)^18^ in three populations experiencing contrasting environmental conditions in terms of climate, resource availability, hunting pressure, and inter-specific competition.

Roe deer provide an especially appropriate model to study habitat selection within individual home ranges because they are highly sedentary and display strong home range fidelity all their life along^19,20^, while being highly selective at a fine spatial scale^21,22^. Moreover, female roe deer are income breeders (sensu Jönsson)^23^ that do not store any body reserve to meet the high energetic expenditures of maternal care^24^. Female roe deer should thus be especially sensitive to variation in resource availability during spring-summer, when energetic costs of late gestation-early lactation peak^25^. Thus, we hypothesize that habitat selection of female roe deer should differ among populations in response to changes of environmental conditions and more specifically to changes of resource availability. We expect that females should not display any functional response in habitat selection when facing highly favorable environmental conditions (i.e., no hunting pressure, high food availability, mild weather conditions). On the contrary, female roe deer should trade cover for food, or food quality for quantity, where environmental conditions are unfavorable due to a high hunting pressure, a low resource availability or quality, or harsh weather conditions, which leads functional responses in habitat selection to occur. Moreover, we expect that female roe deer with similar proportion of each habitat type within their home range but under different environmental conditions should differ in their habitat use. For instance, in good environmental conditions, the use of a given favorable habitat should be independent of its availability. Under such conditions, the cost associated to the time spent searching for a favorable habitat should be compensated by resource availability in this habitat even though such favorable habitats are rare within the home range. Conversely in poor environmental conditions, because of the searching costs of rare resources the availability of a given favorable habitat should reach a certain threshold to be beneficial when used^11^. Such behavior should occur when the cost of searching for a favorable habitat is so high that it cannot be compensated by the benefit associated to the use of resources available in this favorable habitat. Thus, in poor environmental conditions we expect the availability of a favorable habitat to display a threshold beyond which it should be increasingly selected.

## Results

### Estimation of forage quality and quantity

We found large variation in forage quality and quantity among habitat types, but also among the three populations (Table 1). At the Réserve Biologique Intégrale of Chizé (hereafter CH), forage quality and quantity were inversely correlated (Table 1). Shrubs provided high amount of poor quality forage, whereas coppice with standards (hereafter CWS), i.e. mature forest stand, provided a lower amount of high quality forage. Roe deer at CH thus experienced a trade-off between quantity and quality of forage. At La Petite Pierre National Hunting and Wildlife Reserve (hereafter LPP), forage quality remained low and similar among habitat types, but forage quantity was highest in shrubs and in pole stage (made of young trees) than in CWS. Roe deer at LPP should thus select for shrubs and pole stage based on assessment of forage. In the Territoire d’Etude et d’Expérimentation of Trois-Fontaines (hereafter TF), all habitat types had high amount of high quality forage. In this population, roe deer should be weakly selective.

**Table 1:** Mean vegetation biomass (i.e., a proxy of forage quantity), occurrence of hornbeam (i.e., a proxy of forage quality) and forest openness estimated as the horizontal visibility at 25 meters from each habitat patch centroid (i.e., a proxy of protective cover) characterizing each habitat type in the three study areas located in France.

	Forest openness	Chizé		La Petite Pierre		Trois-Fontaines	
		Quantity (g/m^2^) Mean (SE)	Quality (% occurrence)	Quantity (g/m^2^) Mean (SE)	Quality (% occurrence)	Quantity (g/m^2^) Mean (SE)	Quality (% occurrence)
Shrub	<25%	126.6 (78.0)	4.5	95.0 (435.2)	0.5	171.8 (351.3)	24.3
Pole stage	25–50%	114.4 (84.0)	10.8	95.9 (323.2)	0.3	85.1 (163.1)	45.5
CWS	>50%	61.1 (56.9)	12.3	38.7 (252.5)	0.4	135.7 (399.4)	36.6

CWS = coppice with standards.

### Roe deer habitat selection patterns in the three sites

**Figure 1:**
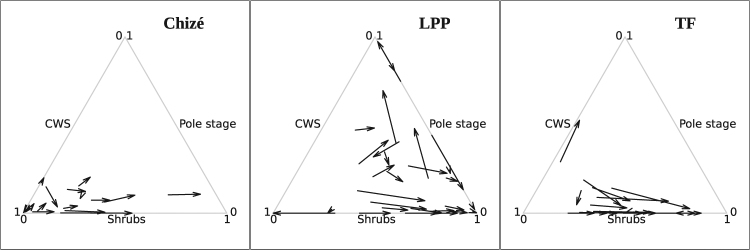
Ternary plots showing the marginality vectors of each female roe deer monitored at Chizé, La Petite Pierre and Trois-Fontaines.

**Figure 2:**
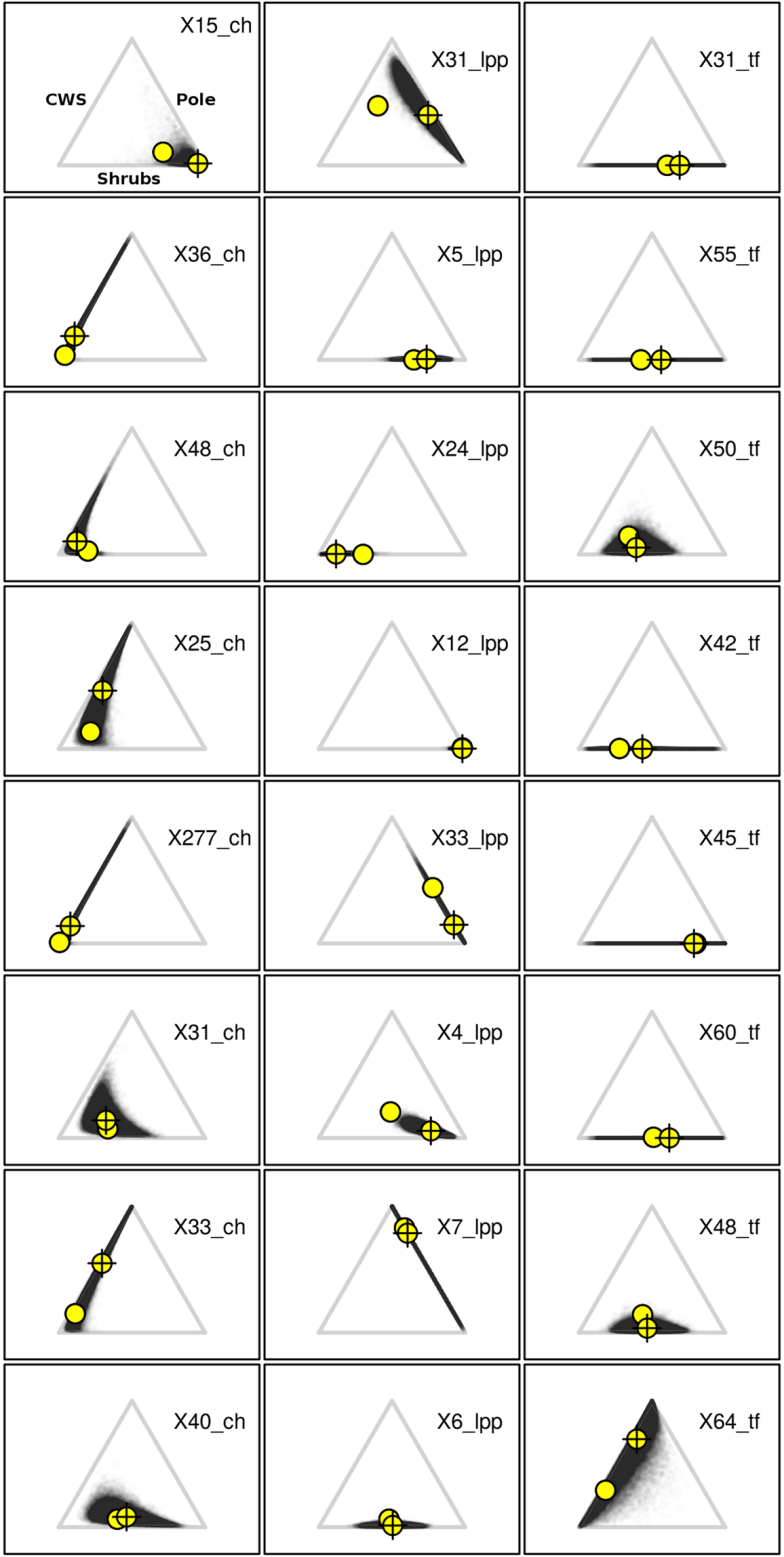
Predicted posterior distribution of the marginality in the ecological triangle for 8 female roe deer selected to be “representative” of the available environmental context experienced in each study area. The left column corresponds to females monitored at Chizé (animal name ending in “_ch”), the central column corresponds to females monitored at La Petite Pierre (animal name ending in “_lpp”), and the right column corresponds to females monitored at Trois-Fontaines (animal name ending in “_tf”). For every possible available home range, we predicted the posterior distribution of the probability of use by calculating, for each vector of parameters sampled by the MCMC, the corresponding predicted vector of proportions of habitat use (each prediction corresponding to a sampled vector of parameter is represented by a translucid grey dot on this plot). For each animal, the distribution of the dots on the ecological triangle represents the distribution of habitat use. The empty circle represents the available habitat within the home range, and the circle with a cross represents the mean of the distribution of habitat use (i.e., mean marginality).

As expected, roe deer habitat selection patterns markedly differed among the three populations (Figs 1 and 2). The model *f2* assuming that habitat selection was different among the three study areas provided a much better fit than the model *f*1 that assumed no difference in habitat selection among the three study areas (difference of DIC = 12, SE = 5). We then used the model *f*2 to predict the average habitat use by roe deer (posterior distribution of the median of the vector ${p}_{s,i}={\{{p}_{s,i,h}\}}_{h=1}^{3}$) for various possible home range compositions in the three sites (Fig. 2). Figure 2 displays the predictions from the selected model for a subset of individuals with representative home range compositions to illustrate the highly variable functional response in relation to the relative availability of the different habitat types in the individual home ranges (see Material and Methods for further information on how these “representative” animals were chosen). Note that the reader can draw this plot for any other animal of our dataset using the function “marginalityDots” of our package “roedeer3sites” provided on github. In particular, see the section 5 of the examples on the help page of this function).

At CH and LPP, the selection of shrubs changed in relation to their availability, which indicated a functional response in habitat selection. Below an availability of 20%, shrubs were avoided (i.e., used less than expected from their availability, as illustrated by the model predictions for roe deer X48_ch at CH and X24_lpp at LPP, Fig. 2), whereas above this availability threshold, roe deer strongly selected for this habitat type (as illustrated by the model predictions for roe deer X15_ch at CH and X4_lpp at LPP, Fig. 2). This shift in selection of shrubs clearly occurred at CH and LPP (Fig. 1). Likewise, roe deer at LPP avoided the pole stage when this habitat type had <50% cover within home ranges. However, when the availability of pole stage increased, we observed a marked variation in selection strength and direction among deer, leading to both a vague general selection pattern at the population level (see observed marginality vectors on Fig. 1), and a large uncertainty in the expected strength of selection (as illustrated by model predictions for the roe deer X31_lpp on Fig. 2). At CH, there was no detectable selection for pole stage: the posterior distribution of the marginality vectors was characterized by a large uncertainty (as illustrated by model predictions for roe deer X33_ch and X277_ch on Fig. 2). Roe deer at TF strongly avoided pole stage when this habitat type was available (as illustrated by model predictions for roe deer X48_tf and X50_tf on Fig. 2), but no clear pattern of selection occurred for the two other habitat types. Shrubs were neither selected nor avoided at TF (as indicated by the large uncertainty of the predicted marginality vectors of our model for roe deer X31_tf and X42_tf on Fig. 2). The examination of the observed marginality vectors (Fig. 1) revealed that the home range composition of three roe deer differed markedly from that of other individuals. By comparing the outcome of the models with and without these three individuals, we safely concluded that these three particular individuals did not influence the results (see the help page of the dataset “coefficientsModel2” in the package “roedeer3sites” provided on github to reproduce this comparison).

### Habitat selection of roe deer sharing similar home range composition in three different sites

**Figure 3:**
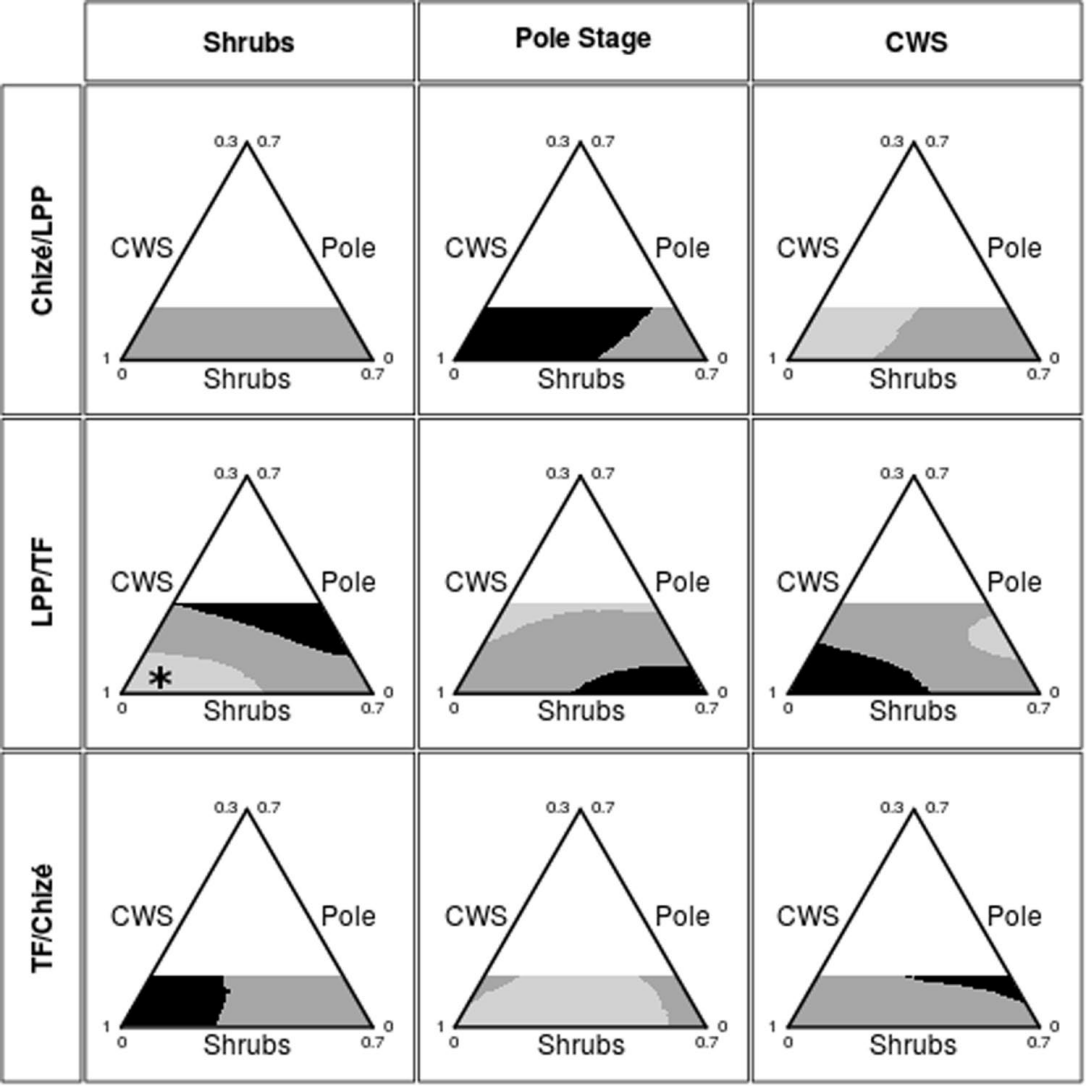
Quantifying the differences in selection of the same habitat type by female roe deer across three study sites in France. For a given pair of study sites (A and B, rows) and a given focal habitat type (*h*, columns), light grey areas correspond to home range composition leading to a more intensive use of the focal habitat type in B than in A (statistical significance is reached when the probability that the use of the focal habitat is greater in B than in A with P > 0.95). Black areas correspond to home ranges where the use pattern of the focal habitat is reversed (i.e. the use of the focal habitat is greater in site A than in site B with P > 0.95). Dark grey areas correspond to home range composition without any detectable difference in the use pattern of the focal habitat between the sites. For example, consider the panel containing the asterisk (comparison of LPP/TF, focal habitat: shrubs). An individual with a home range composition defined by the asterisk (i.e. dominated by CWS and with a small proportion of shrubs and pole stage) would use the shrubs more intensively if it were living in TF than in LPP. Note that for each pair of sites A/B, we assessed the significance of this comparison only for the home range compositions that were possibly available in both study areas A and B, which explain why we did not calculate this probability over the whole ecological triangle (white areas).

To assess the among-study area differences in selection of the different habitat types across their availability, we calculated the log-ratios between the expected use in two areas (for each pair of sites A/B), and calculated the probabilities that this log-ratio was greater than 0, meaning the same habitat selection strength by roe deer in the two areas (Fig. 3). Light grey areas correspond to home ranges where this probability is lower than 5% (indicating that the habitat is more intensively used in the site B put in the denominator of the log-ratio), whereas black areas correspond to cases where this probability is greater than 95% (indicating that the habitat is more intensively used in the site A put in the numerator of the log-ratio). As predicted, the use of a given habitat type did not only depend on habitat availability but also on site (i.e., environmental conditions). Indeed, we found large variation in habitat use among individuals sharing similar home range types in the three populations with contrasting environmental conditions. Consistent with our previous results, no difference occurred in shrub selection between CH and LPP, *i.e*., selection was independent from the environmental context. There was, however, a marked difference between TF and the two other areas. Shrubs were more strongly selected when rare in TF compared to CH and LPP. Inversely, shrubs were more strongly selected when highly available in CH and LPP compared to TF. On the other hand, there was no evidence of different selection of pole stage between LPP and TF, but roe deer avoided available pole stage more strongly at TF and LPP than at CH. Moreover, when pole stage and shrubs were rare, CWS was more strongly selected in LPP than in CH and TF. Finally, when CWS was rare, CWS was more intensively used in TF than in LPP and CH.

## Discussion

Characterising habitat selection and variation in functional response in habitat use of large mammals is becoming increasingly common with the recent surge in GPS-technology^10,11,26^. Most studies report site-specific patterns of selection across a given set of habitat types^16,26^. However, to determine whether such inferences hold across sites, quantifying forage quantity and quality is an important issue. Our direct measures of vegetation cover and forage quality and quantity in each habitat type provided us with a unique opportunity to assess accurately the value of a given habitat type to individuals across various environmental conditions. We were able to show that, even for a given habitat type, forage quantity and quality were highly variable among study areas (Table 1) likely in response to variation in local environmental conditions^27^. Indeed, the comparison of the time spent in each habitat type by roe deer across different populations provided important insight on the mechanisms underlying habitat use and the occurrence of functional responses. We found that female roe deer markedly differed in habitat selection both within and among populations facing various environmental conditions. In the poor overall environmental conditions (CH and LPP), female roe deer clearly displayed a functional response in habitat selection, whereas we detected no functional response in the richest environmental conditions (TF). Such site-specific variation in the occurrence of functional response has previously been reported for moose (*Alces alces*)^11^. However, accounting for variation in environmental conditions provided important insight on the mechanisms underlying the occurrence of functional response.

At CH and LPP, roe deer avoided shrub habitats when they were less common in the home range, but selected for them when they covered more than 20% of the home range. However, the trade-offs generating these functional responses differed between these two study areas. At CH, shrubs provided the largest biomass and CWS the highest quality (Table 1), leading roe deer to face with a trade-off between forage quality and quantity. Consequently, the functional response we observed at CH reflects the compromise for females between the habitat offering the highest amount of forage quantity and the habitat offering the best forage quality to get enough energy to fulfil their need during the critical period of high energetic requirement. Increasing selection for the habitat offering the highest amount of forage at a cost of a reduced use of the habitat including the best forage quality is a common tactic for large herbivores facing poor environmental conditions^13,26^. For instance, Hansen *et al*.^13^ reported that in poor quality areas, female reindeer select more intensively productive habitats with high biomass and plant cover than did reindeer in areas with higher availability of high quality forage. In an income breeder like roe deer, which mostly depends on forage quality during the spring-summer period that corresponds to the critical period of high energetic requirement for females for reproduction^25^, the outcome of such behavior should even be more costly. Indeed, Pettorelli *et al*.^28^ demonstrated that, in CH, females with low quality vegetation in their home range lost their fawns at a much larger extent than other females.

On the other hand, at LPP, shrubs offer both the highest vegetation biomass and the best protective cover, whereas the availability of high quality food resource remains low and similar among the three habitat types (Table 1). Consequently, female roe deer at LPP do not have to trade quality for quantity across spatially segregated resources. In support and contrary to the situation observed at CH, the functional response of habitat use was not related to any measurable food-cover trade-off, nor to a quality-quantity trade-off in LPP, as measured at the landscape level. As proposed by Mabille *et al*.^11^, the observed functional response may arise because roe deer face a trade-off occurring at the lower food patch level spatial scale^13^. Moreover, contrary to roe deer at CH, roe deer at LPP live in sympatry with red deer, and may suffer from inter-specific competition through competition for food^29^. Thus, the outcome of inter-specific competition may provide an alternative explanation to the observed patterns. Indeed, when selecting the habitat with the highest food biomass (i.e., shrubs and pole stage), female roe deer might try to decrease the competition for food resources with red deer^29^ while eventually benefiting from cover to protect them from hunters^30,31^. Such selection for habitat offering cover in areas where animals are heavily harvested is also a common tactic allowing individuals to protect themselves from hunters^16,30,32^. When availability of shrubs in the home range decreased under 20%, roe deer tended to increase selection for CWS with poor cover and low vegetation biomass. Such a strong selection is surprising considering the poor value of this habitat to roe deer (poor protective cover and poor vegetation biomass). Selection for CWS when availability of shrubs is too low could reflect a sub-optimal behavior generated by competition among females, which might be territorial in spring-summer^33^. Moreover, selection for CWS could also result from increased searching costs for shrub patches^30^ or by forage depletion when good habitat patches are too scarce^26^.

Unlike at CH and LPP, female roe deer at TF did not experience any functional response but displayed instead large individual variation in habitat selection. Female roe deer at TF, are not subjected to hunting pressure, and notably benefit from abundant resources of high quality during the critical fawning period. As a consequence, habitat selection should mainly be driven by spatial variation in forage availability. Female roe deer at TF had an easy access to high amount of high quality forage in all habitat types (Table 1). Thus, good environmental conditions experienced by females at TF may account for both the limited selective use of habitat and the high among-individual variation in habitat selection we observed at the home range scale. Under favorable conditions, individuals are expected to be less selective^10,11,34^ and their habitat use should mostly depend on resource availability, which promotes a larger diversity of tactics in habitat use among individuals compared to roe deer living in poorer areas such as CH and LPP^11,13^. Our results suggest that female roe deer are constrained to display a few suitable tactics of habitat use in poor environmental conditions (CH, LPP), which promotes a functional response. On the contrary, in good environmental conditions with no major limiting factors at the home range scale, as it is the case in TF, female roe deer are less constrained by resource availability and can display a higher diversity of space use tactics.

Thanks to the new methodological approach, our study allowed going one step further by comparing habitat use among individuals sharing similar home range composition in contrasted environmental conditions (Fig. 3). Our findings suggest that the way different habitat types or even the same habitat type are used relative to their availability strongly varies according to site-specific environmental conditions. Indeed, we demonstrated that female roe deer sharing similar home range composition adjust selection for a given habitat type depending on the general environmental context of the study site. Female roe deer living in a rich environment such as TF seem to benefit from using a rare habitat, whereas when living in poorer conditions, such as in CH and LPP, females face with stronger limiting factors that led them to use a habitat only when it is abundant above a certain threshold within the home range. Our finding of marked variation in habitat use among female roe deer sharing similar home range composition but facing different environmental conditions suggests that the use of a given habitat type does not only depend on its availability within the home range, but also depends on the general context where it is available. Finally, our results suggests that we could expect a higher diversity of space use pattern in roe deer under highly favorable environmental conditions whereas in constraining environments, roe deer have to cope with trade-off situations and thus demonstrate less space use patterns diversity.

## Material and Methods

### Study areas

**Figure 4:**
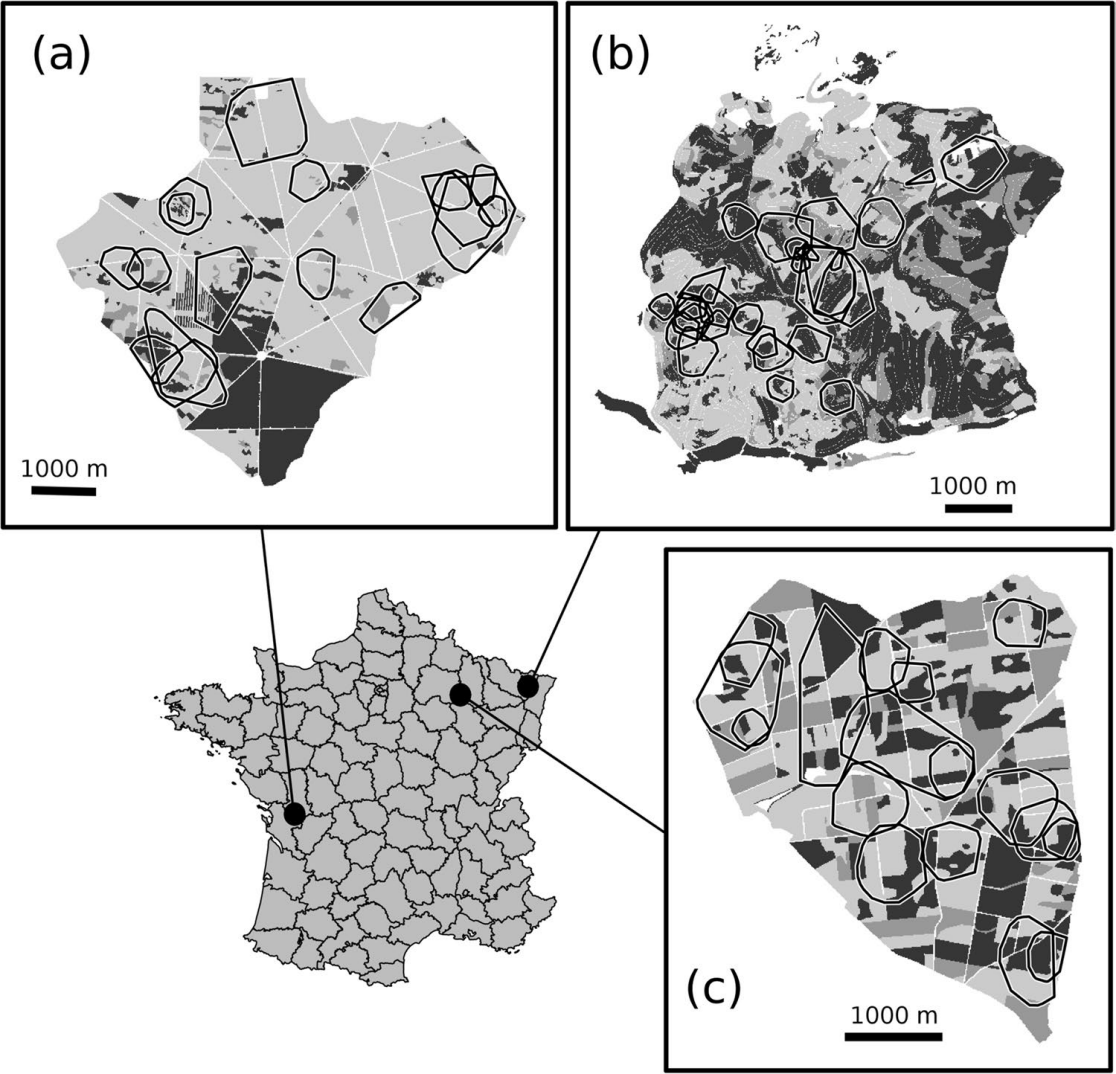
Maps of the three study areas with their location in France: (**a**) Chizé, (**b**) La Petite Pierre, (**c**) Trois-Fontaines. The home ranges of the female roe deer we monitored have been reported on the maps. Three habitat types were defined in each study area: shrubs (dark grey), pole stage (normal grey) and coppice with standards (light grey). This figure was carried out using the R 3.4.0 software (http://www.R-project.org/).

Field work was carried out in three roe deer populations (Fig. 4) in areas that markedly differed in terms of climate, soil composition, forest composition, and forest productivity. Moreover, these populations were subjected to different management regimes. Roe deer were quite heavily hunted all the year long and lived in sympatry with red deer in La Petite Pierre, whereas they were not hunted and lived in absence of red deer in Chizé and Trois-Fontaines.

- The Réserve Biologique Intégrale of Chizé (CH), a 26.14 km2 fenced forest is located in the western part of France (46°05′N, 0°25′W; 47 to 101 m a.s.l.). The climate is oceanic, characterized by mild winters (mean temperature in January of 5.5 °C), hot dry summers (mean temperature in July of 20.5 °C and total rainfall in July-August of 98.0 mm), and frequent summer droughts. The soils are shallow, stony and essentially calcareous under chalky soils in the northern part of the reserve and limestone soils or clay in the southern part^35^. The forest overstory is dominated by oak (*Quercus sp*.) with rich coppices mainly dominated by hornbeam (*Carpinus betulus*) and mapples (*Acer monspessulanum*, *Acer campestris*) in the northern part and a poorer coppice dominated by beech (*Fagus sylvatica*) in the southern part^20^. Productivity of the CH forest is low (long-term average of 3.77 m^3^ of wood produced/ha/year, Inventaire Forestier National), due partly to frequent summer droughts^36^.
- The 26.74 km2 forest of La Petite Pierre (LPP) is located in the Vosges mountains, in north-eastern France (48°58′N, 7°00′E; 300 to 400 m a.s.l.). Climate in LPP is continental, characterized by mild winters (mean daily temperature in January of 0.6 °C) and cool summers (mean daily temperature in July of 18.4 °C and total rainfall in July-August of 175.0 mm). This forest is composed of a mosaic of coniferous and deciduous species including beech, silver fir (*Abies alba*), Norway spruce (*Picea abies*), and Scots pine (*Pinus sylvestris*)^37^. The sandstone substrate produces acidic and poor soils supporting a forest with low diversity and poorly productive (long-term average of 5.78 m^3^ of wood produced/ha/year, Inventaire Forestier National).
- The Territoire d’Etude et d’Expérimentation of Trois-Fontaines (TF), a 13.60 km2 fenced forest located in the north-eastern part of France (48°43′N, 4°56′E; 130 to 230 m a.s.l.). Climate is continental with mild winters (mean daily temperature in January of 2.0 °C) and hot but not dry summers (mean daily temperature in July of 19.0 °C and total rainfall in July-August of 130.0 mm). Forest overstory is dominated by oak and beech with a rich coppice dominated by hornbeam. The soil is fertile and the forest highly productive as indicated by a long-term average of 5.92 m^3^ of wood produced/ha/year (Inventaire Forestier National).

Following the hurricane that hit all the three study areas in December 1999, deep changes occurred in the forest landscape. A substantial proportion of trees fell down in all sites: 19% at CH (4.97 km2, mostly in the beech forest of the southern part), 18% at LPP (4.80 km^2^), and 29% at TF (3.99 km^2^), which led to increase roe deer food availability^38^.

### Roe deer data

All methods were approved by the authorities (French Ministry of Environment). Roe deer captures were performed in accordance to the conditions detailed in the specific accreditation delivered to the Office National de la Chasse by the Préfecture de Paris (agreement n°2009–014). From 2003 to 2008, female roe deer were caught using drive netting (CH and TF) or traps (LPP) between January and March. We equipped only adult females (>1 year of age) with Global Positioning System collars (GPS Lotek 3300 and 3300 S) and released them on site. When possible, we recaptured females previously equipped with collars and replaced the GPS battery to monitor individuals for consecutive years. A total of 61 different females were equipped with GPS collars (17 in CH, 17 in TF and 27 in LPP). Collars were programmed to get a GPS fix every 4 hours all the year long, but we only retained locations collected between April (i.e., the late gestation period) and August (i.e., the rut period) because it corresponds to the most critical life history stage of female roe deer^18^ in terms of energy expenditure (i.e. late gestation and early lactation). We only retained the locations having a positional dilution of precision (PDOP) lower than 5^39^.

### Habitat types

For each study area, we used aerial photographs to identify homogeneous habitat patches (i.e., spatial polygons including a single habitat type). Then we recorded the forest developmental stage in these patches, and digitized them manually into a Geographic Information System^40^. We rasterized maps with a resolution of 10 × 10 m. We first considered four habitat types based on forest development stages: grassland, shrub, pole stage, and coppice with standards (CWS). Forest developmental stages differed in terms of both available vegetation biomass and degree of forest openness (Table 1). The shrub stage corresponds to regenerating stand that provides both good hiding cover and high amount of understory vegetation. The pole stage follows the shrub stage and is made of young trees, which provide less hiding cover than the shrub stage but still include a quite high amount of understory vegetation. Finally the CWS stage corresponds to mature forest stands with poor hiding cover and low amount of understory vegetation. We did not consider the grassland because it only made a small fraction of the home range of the monitored roe deer (36 out of 61 home ranges did not include any grassland at all, and the remaining 25 home ranges only included an average of 4% of grassland [SD = 4.8%]). Thus, we removed the grasslands to infer habitat availability and focused on three habitat types (i.e., shrubs, pole stage, and CWS, Fig. 4).

### Estimation of forage quantity and quality

To quantity forage quality and quantity in each habitat type (Table 1), we first calculated the mean dry biomass per habitat type (g/m^2^), by sampling the number of plant contacts on a 25 × 25 × 165 cm structure (three dimension quadrat) and identified every plant species^41^. Then, we used the hornbeam occurrence as a proxy of habitat quality during the period of maternal care. In addition to be widely distributed in the three study areas, hornbeam is directly related to winter fawn body mass^20^, and provides the best single predictor of fawn survival^28^. At LPP, we used a systematic sampling design, with one sampling plot set every 100 meters, across the forest in May-June in years 2004 and 2005 (1,994 sampling plots). At CH and TF, we sampled biomass every 70 meters within the home range of roe deer females equipped with collars, which led to 930 and 462 sampling plots respectively.

### Selection ratios and marginality vectors

Habitat selection is usually studied by comparing habitat use (U) with habitat availability (A)^3^. To compare A and U we can either focus on additive (U = A + effect) or multiplicative (U = A × effect) effects (see e.g., Cleveland)^42^. When the habitat includes several types, habitat selection is generally studied by working on multiplicative effects. Thus, the most common approach to study habitat selection with several habitat types relies on selection ratios^6^. If *a*_*h*_ defines the proportion of the habitat *h* available to an individual and *u*_*h*_ defines the proportion of time that the individual spends in this habitat type, then the selection ratio characterizing this habitat for this individual is calculated with *w*_*h*_ = *u*_*h*_/*a*_*h*_. The use of these selection ratios is based on a strong theoretical background: under some conditions, they may be proved to be proportional to the probability of selection of a resource unit (e.g., a pixel) characterized by the habitat *h*. In practice this ratio is generally compared to 1 to determine whether the individual exhibits a preference (*w*_*h*_ > 1) or an avoidance (*w*_*h*_ < 1) of the habitat type *h* (e.g., Manly *et al*.^6^). It thus follows that the habitat *h* covering 0.1% of an individual home range, in which this individual spends 1% of its time, has the same selection ratio as the habitat *h’* covering 7% of its home range, where the individual spends 70% of its time. However (i) nothing ensures that if the habitat *h* would have covered 7% of the home range, the preference would have been the same (although this is an implicit assumption when we compare the selection ratios to 1), and (ii) if any other habitat *h”* covers >10% of the home range, habitat selection measured by the selection ratio should necessarily be smaller (because habitat use cannot exceed 100%). Thus, habitat selection measured by selection ratios will typically overemphasize the importance of rare habitat types.

**Figure 5:**
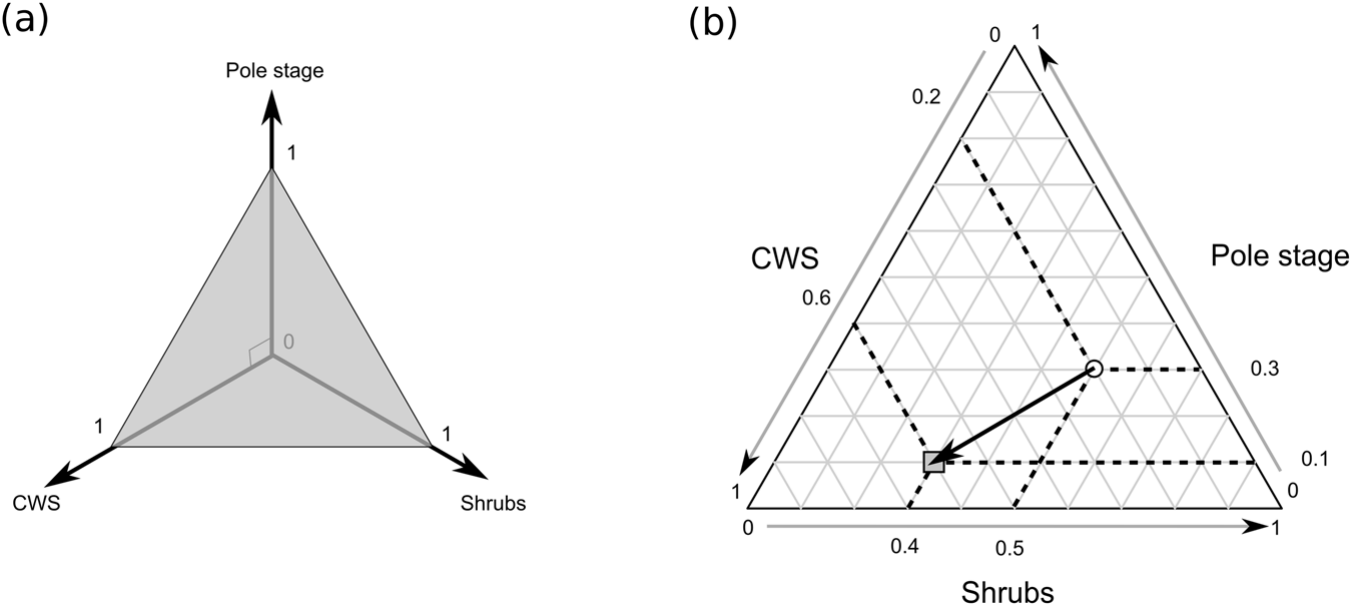
Visualization of the marginality vectors in the ecological space defined by three habitat types. (**a**) Each habitat type defines a dimension in the ecological space. Every habitat composition (whether habitat use or availability) corresponds to a vector of three proportions. This vector defines a point in the ecological space. However, because the cumulative proportions of the three habitat types sum to 1, the possible points are consistently located in a two-dimensional triangular subspace, called ecological triangle in the text (grey triangle). (**b**) Therefore, it is possible to plot every possible habitat composition on this ecological triangle. This ternary plot is an exact representation of the ecological triangle. For illustration, we represented a female roe deer with a home range covered with 50% of shrubs, 30% of pole stage and 20% of coppice with standards (CWS), which spends 40% of its time in the shrubs, 10% in the pole stage and 60% in the CWS. The arrow connecting the available point (white circle) to the used point (grey square) is the marginality vector of the female roe deer.

Although this multiplicative model of habitat selection has already proved to be very useful in many contexts^6^, it may also be of interest to consider additive effects. The habitat type covering 50% of the home range where an individual spends 90% of its time is likely to be more influential on its fitness than the habitat type covering 0.1% of the home range where this individual only spends 1% of its time. Therefore, we considered the marginality rather than selection ratios as a metric for habitat selection (Fig. 5). Marginality has often been used to measure habitat selection on an additive scale^43–45^. The proportion of a given habitat type defines one dimension in the ecological space. The home range of a given roe deer corresponds to the cumulative proportion for each habitat type present in this range, so that every home range is characterized by a point in this multidimensional space. This point describes what is available in terms of habitat types to this individual. Note that because the sum of these proportions is 1, this cloud of “available points” is located in a two-dimensional triangular space (hereafter named “ecological triangle”) when three habitat types are considered, as it is the case in this study. This ecological triangle is embedded within the ecological space defined by the three habitat types (Fig. 5a). The plot of this triangle, named ternary plot^46^, is therefore an exact representation of the ecological space. Similarly, the respective time spent by the individual roe deer in the three habitat types of interest defines a point in the ecological triangle (Fig. 5b). The vector connecting the conditions available to a given roe deer to the conditions used by this individual is the marginality vector. Its length is a measure of the strength of habitat selection and its orientation indicates which habitat types are actually preferred or avoided by a given individual.

### Bayesian model of habitat use

We focused on third-order habitat selection by female roe deer, i.e., on the distribution of the locations of an individual within its home range^3^. Thus, we considered the home range habitat composition as a fixed and known parameter, and we wanted to predict how an individual distributes its locations within the available environmental context in the three study areas. We therefore fitted a Bayesian model of the habitat use by the roe deer to study the differences of habitat selection among the three areas based on the marginality vectors (Fig. 1). Indeed, once we have a reliable model to predict the probability distribution of habitat use as a function of the available habitat types, it is straightforward to use this model to predict the probability distribution of the marginality because (i) the environment available to an individual (i.e., its home range) is assumed to be known and constant, and (ii) the marginality on a particular direction is simply calculated by subtracting this constant habitat availability from the predicted habitat use.

We modeled the use of each habitat type as a function of the whole environmental context available in a roe deer home range using a Bayesian approach. Formally, we fitted the following model:

1 $$\begin{array}{rll}{n}_{s,i} &  \sim  & M({N}_{s,i},\,{p}_{s,i})\\ \mathrm{log}(\frac{{p}_{s,i,h}}{{p}_{s,i,3}}) & = & {f}_{h}({d}_{s,i,1},\,{d}_{s,i,2})+{\varepsilon }_{s,i,h}\\ {\varepsilon }_{s,i,h} &  \sim  & N(0,\,{\sigma }^{2})\end{array}$$

The vector containing the numbers ${n}_{s,i}={\{{n}_{s,i,h}\}}_{h=1}^{3}$ of relocations of the individual *i* from the site *s* in the three habitat types *h* is assumed to be the realization of a multinomial distribution parameterized by the total number of relocations *N*_*s,i*_ of the individual *i* of the site *s* and by the vector of probabilities of use of the three habitat types ${p}_{s,i}={\{{p}_{s,i,h}\}}_{h=1}^{3}$. We modeled the multinomial logit of the probability of use, i.e., the log of the ratio between the probability of use of a given habitat *h* by the individual *i* of the site *s* and the probability of use of a reference habitat type (here taken to be the coppice with standards, *h* = 3). We assumed that the mean multinomial logit of the probability of use of the habitat *h* was a function *f*_*h*_ of the proportions *d*_*s,i,h*_ of shrubs (*h* = 1) and pole stage (*h* = 2) available in the home range – which implicitly accounts for the availability of the coppice with standards (as the three proportions *d*_*s,i,h*_ sum to 1 for a given individual). Note that there was a temporal autocorrelation between successive locations because of the short time lag among successive locations (mean Schoeners’ ratio: 0.5, SD = 0.3)^47^, which could have resulted into an underestimation of the expected variance for the multinomial distribution. We therefore accounted for this autocorrelation by adding a normal over-dispersion residual *ε*_*s,i,h*_ to the model *f*_*h*_.

The use of a habitat type is not only affected by its availability, but also by the availability of the other habitat types: we assume that the whole environmental context affects the selection by animals. We considered two alternative models *f*_1_ and *f*_2_ for the mean multinomial logit of the probability of use. The model *f*_1_ supposed that habitat selection was the same in all sites, i.e., the multinomial logit of the probability of the habitat *h* (with *h* = 1, 2) was modelled as a linear combination of the available proportions of habitat types with coefficients (*a*_*0,h*_, *a*_*1,h*_, *a*_2*,h*_) identical across all sites *s*:

2 $${f}_{1h}({d}_{s,i,1},\,{d}_{s,i,2})={a}_{0,h}+{a}_{1,h}{d}_{s,i,1}+{a}_{2,h}{d}_{s,i,2}$$

The model *f*_*2*_ assumes that habitat selection was different among the three study areas, i.e., the multinomial logit of the probability of the habitat *h* (with *h* = 1, 2) was modelled as a linear combination of the available proportions of habitat types with coefficients (*a*_*0,h,s*_, *a*_*1,h,s*_, *a*_*2,h,s*_) varying among areas:

3 $${f}_{2h}({d}_{s,i,1},{d}_{s,i,2})={a}_{0,h,s}+{a}_{1,h,s}{d}_{s,i,1}+{a}_{2,h,s}{d}_{s,i,2}$$

We defined non-informative prior distributions for all the parameters. The parameters *a = *(*a*_*0,h*_, *a*_*1,h*_, *a*_*2,h*_, *a*_*0,h,s*_, *a*_*1,h,s*_, *a*_*2,h,s*_) involved in the functions *f*_*1*_ and *f*_*2*_ were assumed to be normally distributed *a priori*, and the variance of the over-dispersion residuals was uniformly distributed *a priori*:

$$a \sim N(0,1000)$$

$${\sigma }^{2} \sim U(0,100)$$

We fitted these models with a Monte Carlo Markov Chain (MCMC), using the program JAGS^48^. We built 3 chains of 1,020,000 iterations each (we removed the first 20,000 iterations as a burn-in sample), and we thinned these chains by keeping one sample every 50 iterations. Our approach is closely related to the approach initially proposed by De Valpine & Harmon-Threatt^49^, who introduced the use of the Dirichlet-multinomial distribution to model habitat use with compositional data. However, three main differences between the two approaches can be identified: (i) we modelled habitat use as a function of the whole environmental available context instead of just focusing on the availability of a particular habitat type; (ii) we modelled habitat use in a Bayesian context rather than in a frequentist context to take advantage of the flexibility of MCMC to fit complex models; and (iii) De Valpine & Harmon-Threatt^49^ used the Dirichlet distribution to account for overdispersion, whereas we used a normal overdispersion residual, which is more commonly used to model overdispersion in a Bayesian context^49^.

We used the deviance information criterion (DIC)^50^ to compare *f*_1_ and *f*_2_ and selected the most parsimonious model. We checked the convergence of the MCMC chains both visually and using the diagnostic of Gelman and Rubin^51^. These diagnostics did not reveal any problem of convergence of the MCMC. We examined the fit of the model using the approach recommended by Gelman and Meng^52^. For every value θ^*r*^ of the vector of parameters sampled by MCMC, we simulated a hypothetical replication of the dataset using equation (1), i.e., we simulated a number of locations in each habitat type for each monitored individual. Therefore, for each animal and each habitat type, we simulated 1,000,000 values of the number of locations that could have been observed if this model was true. We then compared the observed number of relocations of each roe deer in each habitat type with the statistical distribution for this number expected under our model (by checking that the observed number falls within the limits of the 95% credible interval derived from this distribution). We found that 99.5% of the 95% credible intervals calculated on these simulated distributions contained the observed number of locations indicating that the fit was correct. Moreover, the examination of the residuals did not highlight any lack of fit of the model.

### Comparison of habitat selection among study areas

We used the best Bayesian model described in the previous section to compare habitat selection among the three areas. We used this model to study the posterior distribution of the marginality vectors predicted by the model for various available home ranges in the ecological triangle. More precisely, for visualization purpose, we considered 8 roe deer chosen for being “representative” of the environmental context occurring in each site. To select these “representative” roe deer in a given area, we built a *K*_*s*_ × *K*_*s*_ matrix (where *K*_*s*_ is the number of individuals monitored in site *s*) containing, at the intersection of the row *i* and of the column *j*, the Euclidean distance measured on the ecological triangle between the available conditions experienced by roe deer *i* and *j*. We then built a hierarchical classification of these animals based on this distance matrix (using the Ward’s minimum variance method)^53^, and we cut this tree into 8 groups (we chose 8 groups based on a visual exploration, which showed that this number of groups allowed illustrating all roe deer selection patterns in the 3 sites). We then randomly sampled one roe deer in each group, and we displayed the posterior distribution of the mean marginality for each individual.

To estimate this posterior distribution for a given individual, we calculated the predicted mean multinomial logit of the probability to use each habitat type from its availability for this individual using equation (1), for the value *r* of the parameter vector ${{\rm{\theta }}}^{r}=({\sigma }^{r},{a}_{0,h,s}^{r},{a}_{1,h,s}^{r},{a}_{2,h,s}^{r})$ sampled by the MCMC. Thus, for a given value of θ^*r*^, it was possible to calculate a predicted vector of habitat use ${\hat{p}}_{s,i}^{r}={\{{\hat{p}}_{s,i,h}^{r}\}}_{h=1}^{3}$. We plotted the distribution of predicted vectors of habitat use on the ecological triangle for each representative individual to visualize the posterior distribution of the marginality.

Finally, for every pair of study areas and every possible habitat type, we used the model to calculate the posterior distributions of the log-ratios:

$$\mathrm{log}(\frac{{p}_{{s}_{1},h}}{{p}_{{s}_{2},h}})$$

For a given habitat type and for a given home range composition, this log-ratio is equal to zero when its use is identical in both study areas *s*_1_ and *s*_2_. It is greater than zero when the use of the habitat type is greater in *s*_1_, and lower than zero when the use of the habitat type is greater in *s*_2_. For every possible home range composition, we estimated the posterior distribution of this ratio (using the same approach used for the estimation of the posterior distribution of the marginality), and calculated the 95% credible interval of this ratio. We could therefore identify the available conditions leading to a greater use of a given habitat type in a given site than in another site.

All analyses were carried out using the R software^54^. We packed the data and R code used for the analysis in the R package “roedeer3sites”, hosted on github at the following URL: https://github.com/ClementCalenge/roedeer3sites. The package “roedeer3sites” can be installed in R with the function install_github() provided in the R package “devtools”, by typing at the command prompt (after installation of the package devtools):

library(devtools)

install_github(“ClementCalenge/roedeer3sites”)

The help page describing the package, accessed by typing help(“roedeer3sites-package”) at the R command prompt, provide detailed information on how to reproduce the results presented in this paper.
